# Towards definitions of time-sensitive conditions in prehospital care

**DOI:** 10.1186/s13049-020-0706-3

**Published:** 2020-01-29

**Authors:** Kristoffer Wibring, Carl Magnusson, Christer Axelsson, Peter Lundgren, Johan Herlitz, Magnus Andersson Hagiwara

**Affiliations:** 1 Department of Prehospital Emergency Care, Region Halland, Sweden; 2 Institute of Health and Care Sciences, Sahlgrenska Academy, University of Gothenburg, SE-405 30 Gothenburg, Sweden; 3 Department of Prehospital Emergency Care, Sahlgrenska University Hospital, Gothenburg, Sweden; 4 Department of Molecular and Clinical Medicine, Institute of Medicine, Sahlgrenska Academy, University of Gothenburg, SE-405 30 Gothenburg, Sweden; 5 Centre for Prehospital Research, Faculty of Caring Science, Work Life and Social Welfare, University of Borås, SE-501 90 Borås, Sweden; 6 Department of Cardiology, Sahlgrenska University Hospital, Gothenburg, Sweden; 7 Prehospen – Centre for Prehospital Research, University of Borås, SE-501 90 Borås, Sweden

**Keywords:** Prehospital care, Time-sensitive conditions, Decision support, Early recognition

## Abstract

**Background:**

Prehospital care has changed in recent decades. Advanced assessments and decisions are made early in the care chain. Patient assessments form the basis of a decision relating to prehospital treatment and the level of care. This development imposes heavy demands on the ability of emergency medical service (EMS) clinicians properly to assess the patient. EMS clinicians have a number of assessment instruments and triage systems available to support their decisions. Many of these instruments are based on vital signs and can sometimes miss time-sensitive conditions. With this commentary, we would like to start a discussion to agree on definitions of temporal states in the prehospital setting and ways of recognising patients with time-sensitive conditions in the most optimal way.

**Main body:**

There are several articles discussing the identification and management of time-sensitive conditions. In these articles, neither definitions nor terminology have been uniform. There are a number of problems associated with the definition of time-sensitive conditions. For example, intoxication can be minor but also life threatening, depending on the type of poison and dose. Similarly, diseases like stroke and myocardial infarction can differ markedly in terms of severity and the risk of life-threatening complications. Another problem is how to support EMS clinicians in the early recognition of these conditions. It is well known that many of them can present without a deviation from normal in vital signs. It will most probably be impossible to introduce specific decision support tools for every individual time-sensitive condition. However, there may be information in the type and intensity of the symptoms patients present. In future, biochemical markers and machine learning support tools may help to identify patients with time-sensitive conditions and predict mortality at an earlier stage.

**Conclusion:**

It may be of great value for prehospital clinicians to be able to describe time-sensitive conditions. Today, neither definitions nor terminology are uniform. Our hope is that this commentary will initiate a discussion on the issue aiming at definitions of time-sensitive conditions in prehospital care and how they should be recognised in the most optimal fashion.

## Background

Prehospital care has changed dramatically in recent decades. This care has evolved from having been a transport organisation where patients were largely transported to the nearest emergency department (ED) to care where advanced assessments and decisions are made early in the care chain. Patient assessments form the basis of a decision relating to the level of care where the patient can a) stay at home with advice on self-care, b) be transported to primary care, c) be included in a care chain for transport directly to a specialist examination or treatment or d) the patient is transported to the nearest ED. This development imposes heavy demands on the ability of emergency medical service (EMS) clinicians properly to assess the patient. To assist in this process, the EMS clinician has a number of assessment instruments and triage systems. Many of these instruments are based on vital signs (VS), such as respiratory rate, oxygen saturation, heart rate, blood pressure and level of consciousness. These can sometimes miss some time-sensitive conditions. Examples of this include patients with a stroke who have normal VS in many cases and decisions must instead be based on symptoms and signs [1]. A prehospital patient safety study found that patients with time-sensitive conditions ran a higher risk of adverse events [2].

It may therefore be of value to define the time-sensitive conditions that are important for EMS clinicians to know and sometimes recognise based on clinical history and examination, regardless of the patient’s VS. These definitions can be used as differential diagnoses in a rule out worst-case scenario (ROW) strategy. They would be of both clinical and scientific value, but there is also a need to define the criteria for raising a suspicion of these time-sensitive conditions in the prehospital setting. These definitions may also improve the EMS clinician’s diagnostic skills. With this commentary, we would like to initiate a discussion on how to agree on definitions of temporal states in the prehospital setting and how to recognise patients with time-sensitive conditions in the most optimal manner.

## Main text

There are several articles discussing the identification and management of time-sensitive conditions. In these articles, neither definitions nor terminology have been uniform. In a systematic review [2] including studies investigating system delays in connection with time-sensitive conditions, these were defined as myocardial ischaemia, stroke, neoplasms, meningitis, dyspnoea, chest pain and transient ischaemic attack (TIA). In a discussion paper [3] on regionalised care for time-sensitive conditions, ST-segment elevation myocardial infarction (STEMI), stroke, cardiac arrest, trauma and intoxication were discussed in connection with regionalisation. The European Emergency Data Project [4] identified five conditions as being time sensitive and having a large socio-economic impact. They were cardiac arrest, respiratory failure, severe trauma, chest pain and stroke. Advanced medical life support (AMLS) is an established concept for the assessment and treatment of non-trauma patients that is well known in both prehospital and hospital emergency care [5]. AMLS uses the terms “life-threatening”, “non-life-threatening/emergent” and “non-emergent” when categorising different chest pain-related diagnoses based on how promptly affected patients require medical care. In connection with chest pain, AMLS classifies tension pneumothorax, pulmonary embolism, oesophagus rupture, acute pulmonary oedema/heart failure, cardiac arrhythmia, aortic aneurysm/dissection, pericardial tamponade and Acute Coronary Syndrome (ACS) as life threatening. This definition of life threatening is fairly comprehensive and useful in many ways in clinical practice. However, heart failure and cardiac arrhythmia are broad diagnoses and they include many conditions with a low risk of death, where immediate care is without importance. In a study performed by the authors [6, 7] with the aim of comparing EMS clinicians’ field assessments with the final diagnoses at discharge from hospital, the following diagnoses were classified as a life-threatening condition: anaphylaxis, myocardial infarction, unstable angina pectoris, TIA/stroke, unconsciousness, septicaemia, aortic rupture, aortic dissection, any form of shock, pulmonary embolism, heart failure including pulmonary oedema, failing heart conducting system, cardiac arrest, intoxication, status epilepticus, obstructive airway, tension/open pneumothorax, cardiac tamponade/contusion, pulmonary contusion, massive haemothorax, flail chest and oesophageal/tracheal bronchial/diaphragm rupture.

Apart from the fact that there is no uniform list of time-sensitive conditions, the terminology is also diverse. Previous studies have used concepts including life threatening, time critical and time sensitive.

There are a number of problems associated with the definition of time-sensitive conditions. For example, intoxication can be minor, but it can also be life threatening, depending on the type of poison and dose. Similarly, diseases like stroke and myocardial infarction can differ markedly in terms of severity and the risk of life-threatening complications. This is further enhanced by the fact that several previous definitions include both symptoms and diagnoses. One example of this is chest pain, which in some cases is caused by time-sensitive diseases, such as aortic dissection and myocardial infarction, but can in many cases have a more or less harmless origin such as gastritis. This is also true of abdominal pain where many conditions can be regarded as non-time sensitive, while others are – for example, a perforating or haemorrhaging peptic ulcer or variceal haemorrhage [8, 9].

There may also be several diagnoses that are missing from the lists that have only been proposals to date. Conducting a modified Delphi process including prehospital and hospital emergency care clinicians, along with representatives of the scientific community may be one way of approaching consensus regarding both terminology and definition. Another approach to reach consensus could be the Utstein-style, which is also a modified nominal group technique.

Apart from the difficulties relating to terminology and definition, another problem is how to support EMS clinicians in the early recognition of these conditions. It is well known that many of them can present without any deviation from normal in VS.

It will most probably be impossible to introduce specific decision support tools for each of the conditions listed above. However, there may be information in the type, localisation and intensity of the symptoms that the patients present [10, 11]. Furthermore, biochemical markers may help to identify patients with time-sensitive conditions and predict mortality at an earlier stage [12–14]. Finally, in terms of myocardial infarction, the use of the prehospital electrocardiogram has been shown to be helpful [15].

Many patients with a time-sensitive condition present with symptoms such as pain, dyspnoea and vertigo. An alternative approach could be to develop a clinical decision support system with machine learning capabilities for all these symptoms, in order to help EMS clinicians to distinguish patients with time-sensitive conditions from those without. For example, machine learning support tools have shown higher sensitivity and faster identification over the telephone in recognising cardiac arrest compared with professional dispatchers [16]. These instruments need to be tested in large patient cohorts in order to prove their eventual value.

## Conclusions

It may be of great value for prehospital clinicians to be able to describe and recognise time-sensitive conditions. Today, neither definitions nor terminology are uniform. We hope that this commentary will start a discussion on the issue in order to aim at appropriate definitions of time-sensitive conditions and their recognition in prehospital care.

## Data Availability

Not applicable

## Abbreviations

ACS: Acute Coronary Syndrome
AMLS: Advanced Medical Life Support
ED: Emergency Department
EMS: Emergency Medical Services
ROW: Rule Out Worst case scenario
STEMI: ST-segment Elevation Myocardial Infarction
TIA: Transient Ischaemic Attack
